# Why does gout target the foot? The pathogenesis of podagra

**DOI:** 10.1186/1757-1146-4-S1-A6

**Published:** 2011-05-20

**Authors:** Edward Roddy

**Affiliations:** 1 Arthritis Research UK Primary Care Centre, Keele University, Staffordshire, ST5 5BG, UK

## 

Gout is the most prevalent inflammatory arthropathy. It displays a striking predilection to affect the first metatarsophalangeal joint (1^st^ MTPJ) as well as joints within the mid-foot and ankle. The propensity of gout for the foot was recognised by the ancient Greeks who named it podagra, literally “foot-grabber”. A number of mechanisms have been proposed to explain this clinical observation. Hyperuricaemia is an essential prequisite for the development of gout, monosodium urate (MSU) crystal formation occurring as serum urate levels exceed the physiological saturation threshold of urate in body tissues. Decreasing urate solubility at cooler distal extremities has been suggested to account for the predilection of gout for the 1^st^ MTPJ, but does not explain why gout targets this joint ahead of the other small joints of the forefoot. *In vitro* mechanical shock leads to formation of MSU crystals and is consistent with the clinical observation that attacks of gout commonly follow minor trauma such as stubbing the toe or physical activity.

More recently, it has been suggested that MSU crystals more readily deposit in osteoarthritic joints. The 1^st^ MTPJ joint is a target joint for osteoarthritis as well as gout, and is the foot joint most commonly affected by osteoarthritis. There is a strong clinical and radiographic association between joints that have been the sites of acute attacks of gout and the presence of osteoarthritis locally at those individual joints. Cadaveric studies demonstrate crystal deposition at the site of degenerate lesions in articular cartilage. Epitaxial MSU formation has also been observed on cartilage fragments. Changes in cartilage and synovial proteoglycans are thought to promote formation and growth of MSU crystals. Transient increases in the urate concentration of resolving synovial effusions owing to the differential permeability of synovium to urate and water, have also been postulated to account for the occurrence of gout at osteoarthritic joints.

In summary, the proclivity of gout for the foot, and the 1^st^ MTPJ in particular, remains poorly understood, and is likely to be multi-factorial, in which the inclination of MSU crystals to deposit in osteoarthritic cartilage is a key component.

